# QTL Mapping for Haploid Inducibility Using Genotyping by Sequencing in Maize

**DOI:** 10.3390/plants11070878

**Published:** 2022-03-25

**Authors:** Benjamin Trampe, Grigorii Batîru, Arthur Pereira da Silva, Ursula Karoline Frei, Thomas Lübberstedt

**Affiliations:** 1 Pivot Bio, 2910 Seventh St, Berkeley, CA 94710, USA; btrampe@pivotbio.com; 2 Department of Agronomy, Iowa State University, Ames, IA 50011, USA; gbatiru@iastate.edu (G.B.); ufrei@iastate.edu (U.K.F.); 3 Stine Seed Inc., 22555 Laredo Trail, Adel, IA 50003, USA; apdasilva@stineseed.com

**Keywords:** maize, haploid inducibility, doubled haploids, genotyping by sequencing, QTL mapping, haploid kernels

## Abstract

Doubled haploid (DH) technology in maize takes advantage of in vivo haploid induction (HI) triggered by pollination of donors of interest with inducer genotypes. However, the ability of different donors to be induced—inducibility (IND), varies among germplasm and the underlying molecular mechanisms are still unclear. In this study, the phenotypic variation for IND in a mapping population of temperate inbred lines was evaluated to identify regions in the maize genome associated with IND. A total of 247 F_2:3_ families derived from a biparental cross of two elite inbred lines, A427 and CR1Ht, were grown in three different locations and Inclusive Composite Interval Mapping (ICIM) was used to identify quantitative trait loci (QTL) for IND. In total, four QTL were detected, explaining 37.4% of the phenotypic variance. No stable QTL was found across locations. The joint analysis revealed QTL × location interactions, suggesting minor QTL control IND, which are affected by the environment.

## 1. Introduction

Inbred line development in maize is one of the most time-consuming tasks in line and hybrid breeding, requiring up to seven to eight generations of self-pollination to achieve acceptable homozygosity [1]. By using in vivo haploid induction and colchicine application for genome doubling, plant breeders can get doubled haploid (DH) lines that are completely homozygous in only two generations, saving substantial time in line development [2,3,4]. 

In vivo haploid production relies on inducer genotypes and requires a relatively high haploid induction rate (HIR) to make the system economically viable [5]. HIRs depend on the one hand on the haploid inducer. Over the decades, haploid inducers with average induction rates of more than 10% have been developed [4,6]. On the other hand, the donor genotype significantly affects haploid induction rates. The ability of different donors to be induced—inducibility (IND), varies significantly depending on the background of the donor germplasm [4,7]. Using the same haploid inducer, HIRs ranged from 0.8 to 33% on average in previous reports [8,9]. 

Increased HIRs significantly enhance the efficiency of DH line production, with particular attention to recalcitrant genotypes [4]. There is only limited information regarding this trait in temperate germplasm. Previous studies reported two quantitative trait loci (QTL) for inducibility with partially dominant effects located on chromosomes 1 and 3, contributing to less than 15% of the phenotypic variation [10]. In addition, the QTL located on chromosome 1 had the largest contribution to phenotypic variation and was located in the same chromosome region as *qHIR1*, which is the major QTL for maternal haploid induction ability [11,12,13], for which the underlying gene *MATRILINEAL* has been isolated [4,14,15].

The objectives of this study were to (i) evaluate the phenotypic variation for IND in a biparental mapping population of temperate inbred lines, and (ii) to identify regions in the maize genome associated with IND.

## 2. Results

### 2.1. Descriptive Statistics and Linkage Map

The average inducibility rate across all locations was 8.26%, with the inducibility rate ranging from 0.63 to 23.76% among families, with plot-based heritability of 0.49 (Table 1).

There was a significant difference in IND across families for all three locations (Table 2).

### 2.2. Single Environment QTL Analysis

A total of four QTL were detected on chromosomes 2, 4, and 5, explaining between 8.1% and 10.7% of the phenotypic variance (Table 3). No QTL were found across all three locations. For percentage data, *qIND2a* has been detected on chromosome 2 at position 180 centimorgans (cM), whereas for angular-transformed data, *qIND2b* was detected at position 202 cM, on the same chromosome. These two QTL were found 22 cM apart. The QTL *qIND4a* was found for percentage data, but not for transformed data (Table 3).

### 2.3. QTL × Environment Interaction Analysis

In QTL × environment interaction analysis for inducibility, four QTL were detected on chromosomes 2, 4, 5, and 8 (Table 4). 

These small-effect QTL individually explained between 5.1% and 9.9% of the phenotypic variance. The QTL on chromosomes 5 and 8 were detected in both analyses, but QTL *qIND2a* and *qIND4b* were only found with percentage data. The *qIND8* was found by QTL × environment analysis, but not identified in the single environment analysis. Only two QTL (*qIND4b* and *qIND8*) showed strong QTL × environment interactions, as indicated by the higher phenotypic variance explained by additive × environment effects (PVE(A × E)) rather than by the phenotypic variance explained by additive effects (PVE(A)) (Table 4).

## 3. Discussion

There was considerable phenotypic variation for IND between families, suggesting a potential for improvement by selection. Moreover, as expected for this trait, the environment and the source germplasm were major factors in the production of haploids (Table 1) [4,16]. In our research, the location effect was highly significant (Table 2). Röber et al. [17] observed variation for means across environments ranging from 2.4% to 22.3%, which is comparable to our results (Table 1). Additionally, it is essential to emphasize that optimization of donor and inducer germplasm along with selection of environments with favorable climatic conditions are likely to positively affect the success of haploid induction [17]. Higher HIR can be obtained by selecting appropriate source germplasm and undertaking pollination under favorable environmental conditions [7], and superior environments can be identified to increase success of induction crosses [4]. 

In contrast to significant location effects, no genotype × environment interactions were detected in our study (Table 2). According to Baye et al. [18], a ‘no’ G–E interaction occurs when one genotype consistently performs better than the other genotype by approximately the same amount across environments. This means all genotypes in our population show similar reaction norms to the environment. Therefore, besides the potential to increase IND over time by either conscious or inadvertent selection in DH breeding programs, there is additional opportunity to further increase IND by understanding environmental factors affecting induction rates.

QTL for low heritability traits are often hard to detect, as well as QTL for highly polygenic traits, even if the traits are highly heritable. In this experiment, the heritability was intermediate with 0.49. De La Fuente et al. [9], evaluating the same trait, observed a narrow-sense heritability of 0.67, whereas Wu et al. [10] observed a narrow-sense heritability of 0.58. In our study, the value for heritability, even though lower compared to previous studies, is still within an acceptable range. In addition, the mapping population in this study was made of a bi-parental cross from two inbreds, whereas previous studies used multiple parents to produce different crosses, which are, consequently, genetically broader.

Because of the high evaluation costs and the time required, there was no adjustment for haploid induction rates based on false positives or false negatives. Moreover, the parents of this biparental cross and a sample of resulting F_2:3_ families could be reliably selected. Since there might be an acceptable misclassification rate [9], depending on the particular population, correction for false positives and false negatives would increase the accuracy and precision of HIR and may thus help to increase heritability, and, consequently, power QTL detection, but at high costs. 

The difficulty in mapping and evaluating HIR is due to a large number of kernels that needs to be evaluated. For example, a total of 1000 kernels would need to be selected to detect a difference of 5%, and smaller differences would require much larger samples [9]. Visual scoring can be a viable selection method, if adequately trained labor is acquired. Nevertheless, issues of misclassification rates are still present. The parents used in this experiment are known to be well selectable with the *R1*-*nj* morphological marker, which is time-consuming and requires extensive labor for evaluation. Other experiments have reported the use of other techniques such as field identification, conventional cytogenetics, herbicide application, and molecular markers [19]. Additionally, others have suggested the use of modern techniques based on fluorescence-based in combination with microspectroscopy and imaging analysis [20]. According to the authors, the potential for high-throughput sorting using fluorescence imaging combined with existing technologies for seed handling make the sorting process viable and a promising alternative. The conventional method based on endosperm and embryo color used in the present experiment is slow and prone to misclassification, which may explain the lower heritability. However, the main goal was to determine the possible presence of major QTL for IND, detectable even with a high misclassification rate. This would have been comparable to a major QTL detected for haploid male fertility in the same mapping population [21], but also to major QTL that have been identified for HIR [6]. However, our and all previous studies did not find evidence for major QTL involved in IND.

Previous results found QTL on chromosome 1 and 3 explaining up to 20% of the phenotypic variation [10]. The QTL found on chromosome 1 by Wu et al. [10] had the largest contribution to the phenotypic variation and was located in the same chromosome region as the qhir1 locus, which is the major QTL for maternal haploid induction. Other experiments suggested that in vivo haploid induction and IND are mainly affected by the male gametes from the inducer lines [22,23,24]. In our case, minor QTL were found on chromosome 2, 4, and 5, explaining together up to 37% of the phenotypic variation, which emphasizes the idea of QTL being population-dependent.

Both data transformation sets identified QTL on the same chromosome with slight differences in the physical position (Table 3). The same was also observed for the QTL × environment interaction, with the exception of the QTL on chromosome 8—which was identified only for the transformed data set. Most QTL mapping methods share the assumption of a normal distribution for residuals. However, there are many situations where this assumption does not hold true (e.g., percentage data, disease resistance, counting). Thus, QTL mapping based on parametric assumptions cannot be directly applied in these cases. On the other hand, least-squares and ANOVA-based methods assume that residual errors are normally distributed [25,26]. Quality of estimation is therefore dependent on the normality of the phenotype.

One approach to guarantee this normality is to attempt to find a mathematical transformation that will convert the trait into a normal variable. The previous study showed that the use of standard QTL mapping methods for the analysis may lead to a significant decrease in statistical power [27], which may explain, why some QTL were only found with transformed data. Consequently, when there is evidence of non-normality, both non-parametric and parametric approaches should be used, as recommended by Kruglyak and Lander [28].

Significant QTL-by-environment interactions were found for qIND8 and qIND4b, for which a significant amount of the phenotypic variance can be explained by additive × environment interactions (Table 4). In preceding studies with barley [29], which estimate and test QTL main effects and Q–E interactions, the main effects appeared to be more important than the Q–E interaction effects for all traits, which means that most detected loci have a constant effect across environments and that Q–E interactions occur independently, regardless of whether the locus has main effects. This is consistent with not finding genotype-by-environment interactions in our results (Table 2). The use of these two QTL would be problematic to use within a breeding program. The breeder would be limited as to when or where the induction of source germplasm could be performed. 

Improvements in IND within the source germplasm can make the in vivo DH system more cost-effective and reduce the costs of haploid selection. IND appears to be highly quantitative in nature. The highly quantitative nature and additive effects of IND indicate that a genomic selection strategy could be used to improve IND in elite germplasm. However, by using the DH process over multiple generations, most likely, inadvertent selection without paying attention to IND will lead to increased IND, because genotypes with higher inducibility are more likely to contribute to the next generation.

## 4. Materials and Methods

### 4.1. Plant Materials

In total, 247 F_2:3_ families were formed from a biparental cross between the inbred lines A427 and CR1Ht. A427 is a public, non-stiff stalk inbred line with expired Plant Variety Protection (ex-PVP), developed at University of Minnesota (GRIN), and CR1Ht is an ex-PVP, non-stiff stalk inbred line developed by the J. C. Robinson Seed Company. Preliminary results showed that CR1Ht had a high inducibility of 16.8%, whereas A427 had an average inducibility of 11.2%. The Iowa State University Haploid Inducers (BHI305, BHI306, BHI307, and BHI310) were bulked due to seed scarcity and used as a maternal haploid inducer (BHI Bulk), as reported previously [21]. The aforementioned inducers are near isogenic inducers with similar induction rates, developed in the backgrounds of inbreds A632.75 and B-15 Dent Sterile, and have inducers RWS and RWK-76 as their source of haploid induction ability. The 247 F_2:3_ families were planted as donor germplasm in three different locations and induced with BHI bulk.

### 4.2. Experimental Design

Field trials were conducted in two different years (2016 and 2017 growing seasons) in isolation fields in Ames, IA. In the 2016 growing season, two locations were used (AM_1_ and AM_2_) in Ames, and in the 2017 growing season, a third location in Ames was used (AM_3_). The locations AM_1_ and AM_2_ were planted in May 2016 and AM_3_ in May 2017. The experiments were planted with four female rows (F_2:3_ families) for every three male rows (BHI Bulk). The BHI bulk was planted at three different planting times to guarantee pollen availability. The experiment was carried out in a randomized complete block design (RCBD) with 3.81 m plots and 0.76 m row spacing. All trials were grown under field conditions using standard agronomic practices. All F_2:3_ families were de-tasseled and open-pollinated by the BHI Bulk.

### 4.3. Phenotypic Evaluation

Inducibility was evaluated on a plot-basis using the *R1*-*nj* seed-based marker system [5]. All of the kernels from each plot were pooled and then sorted into putative haploid and hybrid groupings and counted. IND was calculated as:

(1) IND=Number of haploid seedsTotal number of seeds × 100%

### 4.4. Statistical Analysis

IND was angular-transformed to normalize the distribution of data. Best Linear Unbiased Predictions (BLUPs) were calculated from the proportion and angular-transformed data for the QTL analysis. Data were analyzed using the following model:

Y*_ij_* = μ + E*_i_* + G*_j_*+ ε*_ij_* (2)

where,

Y*_ij_* is the transformed IND rate or IND;
μ is the overall mean;
E*_i_* is the random effect of the *i*th environment;
G*_j_*is the random effect of the *j*th F_2:3_ families;
ε*_ij_* is the residual error.

The variance components and plot mean-based heritability for IND was calculated using SAS PROC MIXED version 9.4 [30]. Entry mean-based heritability was calculated using the formula:

(3) $$h^{2}=\frac{\sigma_{g}^{2}}{\sigma_{g}^{2}+\frac{\sigma_{r}^{2}}{e}}$$

where,

σ^2^_g_—the variance component for genotypes,
σ^2^_r_—the variance component for the residual;
e—the number of environments.

### 4.5. Genotyping

Genotyping of F_2:3_ families was completed using Genotyping-By-Sequencing (GBS) [31]. Plant tissues were collected at the V_2_ growth stage from 10 maize plants per F_2:3_ family and pooled to represent the parental F_2_ individuals. DNA extraction and genotyping were conducted by the Genomic Diversity Facility—Cornell University. For GBS, DNA was digested with the ApeKI restriction enzyme, and DNA fragments were pooled for sequencing using Maize B73 RefGen_v2 and Tassel 5.0 GBS pipeline [32]. A total of 955,690 SNPs were generated for the 247 F_2:3_ families used in IND study.

### 4.6. GBS Correction

TASSEL software version 5 was used to filter GBS data in order to remove single nucleotide polymorphisms (SNPs), with >25% missing data and minor allele frequencies below 5% [32]. A custom R script was created to eliminate SNPs with more than two alleles to avoid potential genotyping errors. TASSEL GenosToABHPlugin converted the SNP nucleotides to a parent-based format [33] and Genotype-Corrector [34] was used to rectify genotyping errors of primarily heterozygous SNP calls from GBS data. A sliding window approach using a window size of 25 SNPs was used to correct genotyping errors and to impute missing data across the genome.

### 4.7. Linkage Map Construction

The linkage map was constructed using 4791 markers on 247 F_2_ plants. The total length of the genetic map was 2090 cM, with a marker density of 2.3 markers/cM across the whole genome. The binning of markers was conducted using the BIN function in QTL IciMapping V4.1 [35]. Chi-square tests were used to identify SNPs with significant segregation distortion for genotypes. Markers with a value of *p* < 0.001 were removed. Linkage map construction was conducted using the MAP function in QTL IciMapping V4.1. The Kosambi mapping function was used to create a linkage map. The traveling salesman algorithm (nnTwoOpt) was used to order the markers. Markers were rippled using the criterion of Sum of Adjacent Recombination Frequencies (SARF) and a window size of five markers. The rippling allows fine-tuning of the marker order to minimize the linkage map length.

### 4.8. QTL Mapping and Analysis for Inducibility

Inclusive Composite Interval Mapping was conducted using QTL IciMapping v4.1 [35]. The BLUPs for phenotypes were calculated in every location, and the QTL-by-environment interaction function was used to evaluate interactions across environments. The LOD threshold was set based on 1000 permutation tests using a Type I error rate of *p* = 0.05. The single environment LOD score threshold was 4.0, and the QTL-by-environment Interaction LOD score threshold was 6.1. Four separate QTL analyses were conducted based on (i) QTL by location, (ii) QTL across locations, (iii) QTL by location using angular-transformed data, and (iv) QTL across locations using angular-transformed data.

## 5. Conclusions

This study revealed considerable phenotypic variation for IND between families, with the environment and the source germplasm as major factors in the production of haploids. A highly significant effect was detected for location, whereas there was no genotype × environment interaction. In addition, the heritability was intermediate, at 0.49. 

Minor QTL were found on chromosomes 2, 4, 5, and 8, explaining together up to 37% of the phenotypic variation, which emphasizes the idea of QTL being population-dependent. Significant QTL-by-environment interactions were found for qIND8 and qIND4b, primarily explained by the additive × environment interactions. The use of these two QTL within a breeding program is not straightforward. Larger breeding programs with multiple locations may be able to exploit such interactions by identifying environments with superior induction results.

## Figures and Tables

**Table 1:** F_2:3_ families phenotypic summary of inducibility (IND) evaluated in three environments. The Mean, Min, and Max values reflect the percentage of haploids.

	AM_1_	AM_2_	AM_3_	Average
Mean	8.04	7.47	9.31	8.26
Min	0.80	0.75	0.63	0.73
Max	18.01	16.61	23.76	19.46
SD	2.91	2.85	3.74	3.27
CV	36.15	38.08	40.17	39.64
Heritability	-	-	-	0.49

SD = standard deviation; CV = coefficient of variation; AM_1_, AM_2_, AM_3_ = location 1, 2 & 3 in Ames, Iowa.

**Table 2:** Analysis of variance for inducibility across three locations using angular-transformed data.

Source	DF ^#^	SS	MS	F-Value	Pr > F
Location	2	0.0445	0.0222	33.09	<0.0001
Families	246	0.4169	0.0017	2.52	<0.0001
Residuals	492	0.3306	0.0007		
Total	740	0.7920			

^#^ DF = Degrees of freedom, SS = Sum of Squares, MS = Mean Square.

**Table 3:** QTL identified in three locations for inducibility using percentage and angular-transformed data.

Percentage Data (%)							
**Env ^1^**	**QTL**	**Chr ^2^**	**Pos ^3^**	**Marker Interval**	**LOD**	**PVE ^4^**	**Add ^5^**
AM1	*qIND5*	5	106	S5.163229787–S5.163889760	5.11	9.1	0.01
AM2	*qIND2a*	2	180	S2.213848716–S2.213842789	4.83	9.5	−0.01
	*qIND4a*	4	119	S4.173795662–S4.174915619	4.11	8.1	−0.01
AM3	*qIND4b*	4	97	S4.158130766–S4.158136040	6.21	10.7	−0.02
**Angular-Transformed Data $\left(\sin^{-1}\sqrt{p}\right)$**							
AM1	*qIND5*	5	106	S5.163229787–S5.163889760	4.95	8.82	8.82
AM2	*qIND2a*	2	202	S2.222648035–S2.222649363	5.19	8.03	8.03
AM3	*qIND4b*	4	97	S4.158130766–S4.158136040	5.48	9.54	9.54

^1^ Environment, ^2^ Chromosome of identified QTL, ^3^ Position of the QTL in cM, ^4^ Phenotypic variance explained, ^5^ Additive Effect (Positive value: increasing allele from A427).

**Table 4:** QTL × environment interactions in three environments for inducibility using percentage and angular-transformed data.

Percentage Data (%)								
**QTL**	**Chr ^1^**	**Pos ^2^**	**Marker Interval**	**LOD**	**PVE ^3^**	**PVE (A) ^4^**	**PVE (A × E) ^5^**	**Add ^6^**
*qIND2a*	2	180	S2.213848716–S2.213842789	7.51	6.24	5.74	0.50	−0.007
*qIND4b*	4	97	S4.158130766–S4.158136040	6.83	9.91	3.55	6.37	−0.005
*qIND5*	5	105	S5.160081320–S5.163229787	7.23	6.50	5.78	0.72	0.004
*qIND8*	4	27	S8.7675588–S8.7748928	6.57	5.97	1.90	4.07	0.003
**Angular-Transformed Data $\left(\sin^{-1}\sqrt{p}\right)$**								
*qIND5*	5	105	S5.160081320–S5.163229787	8.01	6.48	6.06	0.42	0.009
*qIND8*	8	27	S8.7675588–S8.7748928	6.12	5.11	1.65	3.46	0.006

^1^ Chromosome of identified QTL, ^2^ Position of the QTL in cM, ^3^ Phenotypic variance explained, ^4^ Phenotypic variance explained by additive effects, ^5^ Phenotypic variance explained by additive × environment effects, ^6^ Additive Effect (positive values signify that alleles came from A427 and negative values signify that alleles came from CR1Ht).

## Data Availability

Not applicable.
